# Gene mutations and actionable genetic lesions in mantle cell lymphoma

**DOI:** 10.18632/oncotarget.10716

**Published:** 2016-07-19

**Authors:** Makhdum Ahmed, Leo Zhang, Krystle Nomie, Laura Lam, Michael Wang

**Affiliations:** ^1^ Department of Lymphoma and Myeloma, The University of Texas MD Anderson Cancer Center, Houston, Texas, USA; ^2^ The University of Texas Health Science Centre, Houston, Texas, USA

**Keywords:** MCL (mantle cell lymphoma), mutations, actionable genetic lesions, epigenetic, gene targets

## Abstract

Mutations and epigenetic alterations are key events in transforming normal cells to cancer cells. Mantle cell lymphoma (MCL), a non-Hodgkin's lymphoma of the B-cell, is an aggressive malignancy with poor prognosis especially for those patients who are resistant to the frontline drugs. There is a great need to describe the molecular basis and mechanism of drug resistance in MCL to develop new strategies for treatment. We reviewed frequent somatic mutations and mutations involving the B-cell pathways in MCL and discussed clinical trials that attempted to disrupt these gene pathways and/or epigenetic events. Recurrent gene mutations were discussed in the light of prognostic and therapeutic opportunity and also the challenges of targeting these lesions. Mutations in the *ATM, CCND1, TP53, MLL2, TRAF2* and *NOTCH1* were most frequently encountered in mantle cell lymphoma. Translational models should be built that would assess mutations longitudinally to identify important compensatory, pro-survival and anti-apoptic pathways and actionable genetic targets.

## INTRODUCTION

Mantle cell lymphoma (MCL) is a B-cell, non-Hodgkin's lymphoma (NHL) with poor prognosis. Newer approach to treatment largely focused on modulating the B-cell signaling pathways to inhibit tumor proliferation [1–5]. A multitude of drugs have been developed and also under development that target specific B-cell signaling pathway components. The spleen tyrosine kinase (SYK) inhibitor, Bruton's tyrosine kinase (BTK) inhibitor, phosphoinositide-3 kinase (PI3K) inhibitor and the mammalian target of rapamycin (mTOR) inhibitors have been studied actively in pre-clinical settings and in clinical trials. Of these, BTK inhibitor ibrutinib showed durable efficacy in relapsed MCL as a single agent and currently considered drug of choice in the treatment of relapsed/refractory cases [6]. However, acquired and primary resistance to ibrutinib has caused disease progression [7, 8]. As increasing number of patients are being treated with the frontline agents including but not limited to ibrutinib, drug resistance is emerging as a bottleneck in the treatment of and potential cure for MCL. There is a great need to overcome resistance and developing new strategies to improve clinical outcome of these patients. Genetic alterations might hold the key to understanding treatment refractoriness.

The objective of this review was to identify frequent gene mutations in MCL, discuss their potential as therapeutic targets and summarize the current state of knowledge about the clinical trials that affect these mutations through therapeutic agents. Future directions on how to utilize genetic aberrations in treating MCL is also discussed.

## SOMATIC MUTATION AND EPIGENETIC EVENTS

Somatic mutation plays a very important role in transforming normal cells to cancer cells, regardless of the tissues affected or age of the patients[9]. Apart from somatic mutation, the epigenetic events such as chromatin modification and DNA methylation have also been recognized as key elements in cancer progression [10–12]. Since MCL is a B-cell lymphoma, the B-cell signaling pathways downstream from receptor activation is crucial in understanding mechanism of drug resistance. Mutations could affect the involving pathways either at a receptor level or downstream. This raises the possibility of cancer cells evading therapeutic agents that target specific B-cell pathway components. With the advent of next generation sequencing (NGS) technologies through whole genome sequencing (WGS), whole exome sequencing (WES) and miRNA expression profiling [13, 14], studies are increasingly reporting molecular alterations among cancer cells. This provides a great tool to profile gene mutations in MCL and to explore the possibility of using the mutations as prognostic variables. The mutation profiling could potentially increase the probability of therapeutic success. Understanding the prevalent gene mutations in MCL might provide insight into the complex interplay of genes; their proteins and how to possibly overcome drug resistance.

## DIVERSE PROGNOSIS OF MCL AND THE NEED FOR “PERSONALIZED MEDICINE”

The clinical prognosis of MCL is a diverse spectrum which could be explained by the genetic heterogeneity of the tumor. At one end we have indolent MCL while others suffer from very aggressive refractory disease. Among younger patients, high dose chemotherapy followed by autologous stem cell transplant (ASCT) is considered the standard consolidation therapy [15, 16]. However, majority of MCL patients are older and acute and late toxicities associated with high dose chemotherapy question the appropriateness of such approach. The mantle cell lymphoma international prognosis index (MIPI) stratifies MCL patients into low, intermediate and high risk groups based on clinical prognostic factors [17]. However, genetic mutations such as *TP53, CCND1* have been reported as important molecular markers that has prognostic value and could improve the MIPI index [18–20]. The mutational information could correlate with treatment outcomes providing the opportunity of “personalized medicine” for patients at different spectrum of the disease. Greater understanding of genetic mutations at diagnosis and following treatment could potentially allow altering treatment path, essentially guiding clinical practice in MCL in future.

## RECURRENT SOMATIC/EPIGENETIC MUTATIONS IN MCL

The somatic mutations and epigenetic lesions in MCL are summarized in Table 1. We reported 11 mutated and/or deleted genes with rank orders in terms of the most to the least frequencies as reported by six studies that described somatic/ epigenetic mutations in MCL. A total of 552 patients samples' were used for mutational analysis by all six studies. Bea et al. [21] described somatic mutations in 29 MCL cases followed by a targeted sequencing of an independent cohort of 172 MCL patients. Twenty of 29 (69%) of the samples were collected at diagnosis while 21% were collected pre-treatment and 10% at progression/ relapse of the disease. Eighty percent of the patients were at Ann Arbor Stage IV and most (60%) of the samples were collected from peripheral blood and about 30% from lymph nodes [21]. The most frequently mutated gene that Bea et al. [21] identified was *ATM (41.4%), CCND1 (34.5%), TP53 (31%), MLL2 (14%), WHSC1 (10%), BIRC3 (6%), NOTCH2 (5%), NOTCH1 (5%), MEF2B (3%)* and *TLR2 (1%)*. Similar frequency of specific mutations was observed by Greiner et al. [22]; they found mutated and/or deleted *ATM (40%)* and *TP53 (26%)* in MCL cases who were cyclin D1-positive among a cohort of 92 untreated patients [22, 23]. The genetic landscape of MCL has been described by Zhang et al. [24] among a cohort of 56 cases of which 44 (78.5%) tumor samples were collected from lymph nodes with 28 (50%) corresponding normal tissue, mostly from bone marrow. Zhang et al reported *ATM (42.8%), CCND1 (16%)* and *RB1 (10.7%)* as the most frequent mutations in MCL. They included a comprehensive list of 37 mutated genes identified by the study although we included mutations that are reported by more than one study in Table 1. Apart from these somatic genes reported above, the *MLL3* (16%) and *TRAF2* (14%) are reported by two separate studies to be frequently identified in MCL cases [24, 25]. The *MLL* is epigenetic modifier whereas the *TRAF2* is a somatic gene involved in the nuclear factor kappa beta (NF-KB) pathway which is discussed below.

**Table 1:** Recurrent gene mutations among patient cohorts and their frequencies in mantle cell lymphoma

Rank order #	Genes	Functional group	Bea et al[21], MCL cohort, *N* = 29	Greiner et al[22], MCL cohort, *N* = 92	Zhang et al[24], MCL cohort, *N* = 56	Rahal et al[25], MCL cohort, *N* = 165	Kridel et al.[26] MCL cohort, *N* = 108	Meissner et al[27], MCL cohort, *N* = 102	Rossi et al. [32], MCL cohort, *N* = 151
1	*ATM*	Somatic/cell cycle	41.4%	40%	41.9%	49.5%		50%	42%
2	*CCND1*	Somatic/cell cycle	34.5%		16%	19%	19%	18.6%	14%
3	*TP53*	Somatic	31%	26%	19.6%	14%		13.7%	7%
4	*MLL2*	Epigenetic	13.8%		19.6%				12%
5	*MLL3*	Epigenetic			16%	16%			
6	*TRAF2*	Somatic				7%			1%
7	*NOTCH1*	Somatic/development	4.6%		14.2%	14%	12%	13.7%	6%
8	*WHSC1*	Epigenetic	10%		7%				13%
9	*BIRC3*	Somatic/cell adhesion	6.4%		8.9%	8%			5%
10	*NOTCH2*	Somatic	5.2%						
11	*UBR5*	Ubiquitin-proteasome			7.1%	18%		17.6%	

Among a cohort of 108 patients, 92% of whom were biopsied at diagnosis, Kridel et al. [26] reported *NOTCH1* mutation in 12% of the clinical samples. Similarly, Meissner et al. [27] reported frequency of somatic mutation among a cohort of 102 patients with 94% samples collected at diagnosis, 79% of the patients at stage III or IV (Table 1). They reported *UBR5* mutation among 18% of the patients which were not described in MCL prior to this study. *UBR5* could play a crucial role in the pathogenesis in MCL as it has roles in DNA damage response, cell cycle control in addition to E3 ligase function [28, 29]. Among the *CCND1* positive patients, the prevalence of *MYC* and *BCL-2* aberration were reported as 36% and 24% with only *MYC* being the independent factor for poor survival [30]. These mutations could have great implications in the prognosis in MCL as patients with primary resistance to ibrutinib were reported to be more likely to express novel mutations [31].

Taking the cue from previously reported somatic mutations, Rossi et al [32] explored the clinical relevance of recurrent mutations in MCL. They performed deep sequencing analysis of a panel of genes (*ATM, CCND1, TP53, MLL2/ WHSC1, BIRC3, TRAF2 and NOTCH1*) in a prospective phase III trial. In their preliminary data, they concluded that young patients may benefit from the combination of two genetic biomarkers, *TP53* and *MLL2* (also known as *KTMD2*) in a cytarabine-based chemotherapy followed by ASCT. In their data, even intensive chemotherapy was not able to reverse the negative prognostic impact of *TP53* mutation.

## MUTATIONS INVOLVING THE B-CELL SIGNALING PATHWAYS IN MCL

When the receptors presents on the membrane of the B-cells bind to external ligands, a complex network of intracellular signaling pathways ensues. The function and survival of the B-cell is largely dependent upon these pathways which are interconnected. When a somatic mutation involves the signaling pathway, there is disruption to the regulation of the cell activity. Several B-cell signaling pathways are implicated in the pathogenesis of MCL. However, we will be focusing on relevant pathways that were reported to harbor mutations in MCL. Five main pathways were described in the B-cell signaling that are targets for somatic mutations: the B-cell receptor (BCR) pathway, the toll like receptor (TLR) pathway, the NOTCH signaling pathway, Nuclear factor kappa-beta (NF-KB) pathway and the mitogen activated protein kinase (MAPK) pathway[33]. Although, there were quite a few genes that were found to be mutated in several B-cell neoplasms, in MCL the *BIRC2/BIRC3*, the *TRAF2/TRAF3* and *NOTCH1* are the somatic mutations described to date that affect the B-cell signaling pathways (Table 2).

**Table 2:** Mutations involving the B-cell pathways in MCL

Gene	Site/pathway	Result of mutation	Associated somatic mutation	Potential therapeutic strategy
*C418S*	BTK	Persistent activation of BTK and AKT (ibrutinib resistance)	*CCND1*	Palbociclib (inhibition of CDK4) plus Ibrutinib
*NOTCH1*	PEST domain/NOTCH pathway	Phosphorylation and ubiquitylation of multiple sites of NOTCH intracellular domain; activation of transcription of downstream genes		
*BIRC2*	Somatic/NF-KB pathway	Activation of the alternate NF-KB pathway (possible mechanism of ibrutinib resistance)		
*BIRC3*	Somatic/NF-KB pathway	Activation of the alternate NF-KB pathway and direct effect on cells (possible mechanism of ibrutinib resistance)		
*TRAF2/TRAF3*	Somatic/NF-KB pathway	Activation of the alternate NF-KB pathway (possible mechanism of resistance)		
*NIK (MAP3K14)*	BCR pathway	Activation of the alternate NF-KB pathway (possible mechanism of ibrutinib resistance)	*BIRC2/TRAF3*	Ibrutinib plus NIK inhibitor

The mutations in *TRAF2 or BIRC3* are associated with resistance to ibrutinib therapy. As B-cell receptor (BCR) modulator, ibrutinib indirectly inhibits the classical NF-KB pathway. However, the *TRAF2* mutant cells were reported to depend on the alternate NF-KB pathway which is, inturn, dependent on the activation of the protein kinase *NIK* (also known as *MAP3K14*). Rahal et al. [25] described the *NIK* dependency of the *BIRC3* or *TRAF2*. In essence, the loss of function of either of these two genes (*TRAF2 or BIRC3*) due to mutation leads to the *NIK* activity. Because of this dependency there is potential for *NIK* as a therapeutic target among those patients who are resistant to BCR pathway signaling inhibitors such as ibrutinib. To date, we do not have a potent inhibitor of *NIK* in clinical settings although work is underway for discovering compounds with potent activity[34]. In multiple myeloma (MM) cell lines, two *NIK* inhibitors were able to demonstrate selective toxicity for cells that had mutations that activate the alternate NF-KB pathway[35]. In the same MM cell lines, cells with mutation in classical NF-KB pathway were not affected by the *NIK* inhibitors demonstrating the *NIK* dependency of the alternative NF-KB.

The *BIRC3* mutation mentioned above has the potential for direct transformation to cancer cells independent of the NF-KB signaling[36]. The *BIRC2/BIRC3* mutated cells lack the RING finger domain; and there could be molecular targets that mediate these somatic mutations driven carcinogenicity which is yet to be described. As a result of this potential direct effect of the *BIRC3* mutation, the approach to overcome ibrutinib resistance through a combination of *NIK* inhibitor and BTK inhibitor could lead to treatment failure among those patients that harbor the somatic mutation. Future works should assess the treatment response among patients who have genetic lesions in *TRAF2/BIRC3* and those who do not harbor mutation in these genes.

The *C481S* gene located at the binding site of BTK has been described as the first relapse-specific secondary gene mutation [37]. Because of the mutation, ibrutinib's affinity to the enzyme is weakened which led to ineffective BTK inhibition. In the persistent BTK and AKT/mTOR pathways which were secondary to the mutation, the CDK4 mediated cell proliferation occurred. PD 0332991 known as palbociclib which is a CDK4 inhibitor was able to induce prolonged G1 arrest in such cells. Thus, those patients with the mutated BTK (*C481S)* would benefit from a combination of ibrutinib and CDK4 inhibitors.

## MUTATIONS AND CLINICAL TRIALS IN MCL

Table 3 summarizes the clinical trials that used drugs that involve the mutated genes among MCL patients.

**Table 3:** Genes implicated in clinical trials involving MCL patients

Genes implicated	Trial type, reference	Intervention/regimen	Findings	Cancer types, MCL (n)
*ATM*	Phase I[40]	Cyclophosphamide +Veliparib	Prolonged stable disease (>6 cyc.)	SLL (*n* = 2)
*BCL-2*	Phase I[72]	ABT-199	ORR 48%	NHL/MCL = 12
*CCND1*	Phase I[47]	Bortezomib+ Alvocidib	33% total response rate for NHL and progressive disease (PD) in MCL	NHL/MCL (*n* = 3)
*CCND1*	Phase I[49]	Flavopiridol	PR in MCL	NHL/MCL (*n* = 2)
*CCND1*	Phase II[50]	Flavopiridol	PR 3.3 months (*n* = 3); SD 3.4 months (*n* = 20); PD (*n* = 5). Overall response with no prior therapy is 11% vs. 6% with prior therapy	MCL (*n* = 28)
*CCND1*	Phase I[51]	Flavopiridol + Fludarabine + Rituximab	80% ORR for MCL (n = 10)	NHL/MCL/CLL
Epigenetic modifiers	Phase II[57]	Vorinistat + Rituximab	33% PR/ORR for MCL (M = 3)	NHL/MCL (*n* = 3)
Epigenetic modifiers	Phase II[55]	Vorinostat	ORR for MCL 0-60% (*n* = 4)	NHL/FL/MCL (*n* = 4)
Epigenetic modifiers	Phase I[58]	Panobinostat + Everolimus	100% PR for MCL (*n* = 3)	HL/NHL/MCL (*n* = 3)
Epigenetic modifiers	Phase II[56]	Vorinostat	OS 16.9 months for MCL (n = 9); SD 1/9	FL/MCL (*n* = 9)/MZL
Epigenetic modifiers	Phase I[54]	Vorinostat	SD (n = 1) & CRu (*n* = 1) for MCL	DLBCL/CTCL/MCL (*n* = 2)/FL
Epigenetic modifiers	Phase I[59]	Vorinostat + RICE	Sensitive 60% (*n* = 5) MCL	FL/MZL/DLBCL/MCL (*n* = 5)
Epigenetic modifiers	Phase I/II[61]	Abexinostat	ORR 27.3%; median PFS 3.9 months for MCL	FL/MCL (n = 11) (for phase II)

SLL=small lymphocytic lymphoma; NHL= non-Hodgkin's lymphoma; FL=follicular lymphoma; MCL= mantle cell lymphoma; MZL=marginal zone lymphoma; DLBCL= diffuse large B-cell lymphoma; CTCL= cutaneous T cell lymphoma

### ATM

This genetic lesion is the most frequent mutation described among MCL. The gene, known as the *Ataxia Telangiectasia Mutated (ATM)* is a tumor suppressor gene which encodes a protein that signals DNA damage. The role of *ATM* mutation alone is debated in MCL and it was reported that *ATM* mutation, on its own, may not affect patient's overall survival [22, 32]. However correlation of *ATM* with *TP53* aberration could lead to important phenotype. As a result of the *ATM* mutation, cells have impaired apoptosis and defects in the double strand break (DSB) repair[38]. The intermediary in the DNA damage-repair process is the enzyme poly ADP-ribose polymerase (PARP) which recruit proteins to the damaged sites of the DNA[39]. This provided the opportunity for therapeutic intervention of the enzyme by PARP inhibitors in *ATM* deleted lymphoid tumors. Olaparib and veliparib are two PARP inhibitors that have been used either alone or in combination with other chemotherapeutic agents and enhanced cyto-toxicity in *ATM* deleted MCL cell lines by PARP inhibitors were reported [40, 41]. We did not find any clinical trials among MCL patients that used PARP inhibitors. However, a phase I trial of veliparib was not beneficial among two patients with small cell lymphoma [40]. Another phase I trial of PARP inhibitor CEP-9722, Gemcitabine and Cisplatin has been completed recently but no study results are posted yet (NCT01345357).

Cells with *ATM* deletion/ dysfunction show increased radio-sensitivity. This is potentially important as a therapeutic strategy in treating MCL. In clinical practice, fear of radiotoxicity in normal tissue resulting from *ATM* inhibition limits the use of radiotherapy among patients who have dysfunctional *ATM.* In an innovative experiment, Moding el al. showed that ATM deletion preferentially radio-sensitizes the tumor endothelium not affecting normal cardiac endothelium in the mice model [42, 43]. By using the stereotactic body radiation therapy (SBRT), in which relatively high dose of radiation (40-60 Gy) is delivered in 1-5 fractions, the tumor cells can be targeted with a curative intent as described by Moding et al[44]. While this body of work is in the mice model, data from MCL patients indicate that radiation is indeed an effective treatment modality even among heavily pretreated and chemo-refractory patients [45]. Furthermore, unlike sarcoma MCL is a radiosensitive tumor and a low dose of radiotherapy is sufficient. Therefore, using radiotherapy with a curative intent in *ATM* mutated MCL patients is an attractive strategy.

### CCND1

Cyclin D1 is a member of a family of three types of cyclin genes which is not expressed in normal B-cells. The epigenetic event in the pathogenesis of MCL is the common translocation t(11;14) which leads to the deregulated expression of the cyclin D1 protein and proliferation of B lymphocytes[46]. The cyclin D1 protein works in conjunction with cyclin dependent kinases (CDK) such as the CDK4/ CDK6. The CDK inhibitors such as flavopiridol have been tried in MCL either alone or in combination with the proteasome inhibitor bortezomib [47–51]. A natural flavonoid found in turmeric called curcumin has been shown to down regulate the *CCND1* in MCL cells lines [52].

In a single agent flavopiridol phase I trial among Non-Hodgkin's lymphoma (NHL), only two patients with MCL showed partial response [49] (Table 3). A phase II trial with larger sample size for MCL (*n* = 28) conducted in Canada with single agent flavopiridol did not show promising outcome. The overall response with no prior therapy was only 11% (6% with prior therapy), 23 (82%) of the patients had either partial response or stable disease for 3.4 months [50]. When combined with fludarabine and rituximab, MCL patients (*n* = 10) had 80% ORR [51]. A combination of flavopiridol and bortezomib achieved no response in MCL[47]. PD 0332991 (palbociclib)- another flavopiridol that inhibits the CDK4, has been shown to cause synergistic induction of PIK3IP1 and inhibit the PI3K-AKT pathway activation which overcomes the acquired mutation and resulting resistance to ibrutinib[37, 53].

### Epigenetic modifiers (MLL2/MLL3)

The histone deacetylase inhibitors (HDAC) are newer agents that have been used among MCL patients. These agents remove the acetyl group from the histone protein and inhibit the HDAC enzyme that regulate the transcriptional and post translational processes[4]. We reviewed seven clinical trials that used HDAC inhibitor either alone or in combination with rituximab or everolimus. Two MCL patients in a phase I study of vorinostat showed stable disease (SD) and complete remission (CR) (unconfirmed)[54]. In a phase II study in Japan, the efficacy of vorinostat (HDAC inhibitor) alone could not be estimated (the overall response could be between 0-60%)[55]. For refractory or relapsed cases of MCL (*n* = 9) the OS was reported to be 16.9 months for single agent vorinostat with SD in only one patient [56]. Vorinostat, in combination with rituximnab achieved 33% overall response rate (ORR) among MCL patients (*n* = 3) in a different phase II trial in California, U.S[57]. Panobinostat, another HDAC inhibitor showed 100% partial response (PR) in combination with everolimus among three patients with MCL[58]. When combined with the RICE regimen, vorinostat was 60% sensitive to MCL cells from five patients[59]. Another single agent HDAC inhibitor, abexinostat only achieved 15% ORR for MCL in the preliminary results of an ongoing trial[60]. In a phase I/II trial, Evans et al [61] reported 27% ORR for single agent abexinostat among a cohort of 11 MCL patients.

### TP53/NOTCH1/BIRC3/TRAF2

As mentioned before, the *TP53* and *ATM* are interrelated; both are involved in the regulation of apoptosis and cell cycle. Targeting the wild type *TP53* by inhibiting the endogenous regulator HDM2 and also by non-HDM2-mediated peptide inhibitor has been tried in solid tumors [62, 63]. We did not find any clinical trial among MCL patients involving these agents.

For *NOTCH1* mutation, γ-secretase inhibitor was used in T-cell lymphoblastic leukemia but not in MCL[64]. Similarly, we did not find clinical trials among MCL patients targeting the *BIRC3*, or *TRAF2/TRAF3* mutations.

### BCL-2

*BCL-2* is an anti-apoptic protein and which is highly expressed in NHL. In an ongoing phase I study (NCT01328626), *BCL-2* inhibitor ABT-199 showed notable response as a monotherapy in MCL. The ORR was 48% for nine MCL patients with one complete remission[65]. Among 56 patient-samples in cell cultures as well in the xenografted MCL, targeting *BCL-2* by ABT-199 resulted in synergistic growth inhibition with ibrutinib [66]. Similar synergistic growth inhibition and induction of apoptosis was reported by Zhao et al[67] when ibrutinib was combined with ABT-199 in MCL cells. Indeed, MCL cell lines and primary MCL cells were highly sensitive to *BCL-2* inhibitor (ABT-199) regardless of ibrutinib sensitivity[68]. It is possible that in presence of ibrutinib resistance, *BCL-2* is up-regulated; thus targeting this could be an alternative therapeutic strategy for a subset of MCL patents. High response rate of ABT-199 as a single agent in relapsed/refractory cases were reported in CLL and its combination with other agents such as rituximab and bendamustine has achieved impressive ORR in CLL[69]. Thus inhibiting *BCL-2* could lead to very important treatment breakthroughs in multiple hematological malignancies including MCL.

## DISCUSSION AND FUTURE DIRECTIONS

We reviewed literature published in the last 10 years that describe recurrent gene mutations in mantle cell lymphoma and their role in the treatment bottleneck of drug resistance. We identified from studies that conducted next generation sequencing of MCL patients' samples that *ATM*, *CCND1*, *MLL2, TP53* and *BIRC3* are among the most frequent recurrent somatic gene mutations in MCL. One important translational feature is that by stratifying patients at diagnosis based on their *TP53* and *MLL2* mutational status, the benefit of a specific therapy (cytarabine-based chemotherapy) could be predicted among young patients[32]. Similar works need to be done to validate patient-stratification strategies based on tumor mutation among other patient populations. Of the somatic mutations that we reviewed, *ATM* kinase mutation was the most frequent. This has interesting translational potential of targeting *ATM* deleted/ mutated MCL cases with low dose radiotherapy (RT). RT has been effective in indolent MCL and also among relapsed/refractory cases [45]. Combining RT along with targeted therapy might provide durable remission. We have reviewed the *ATM* mutation and radio-sensitivity in MCL and the therapeutic potential of RT elsewhere.

For the B-cell signaling pathways, we reviewed mutation in the BTK enzyme *(C481S)*, *NOTCH1, TRAF2* and *BIRC3*. Of these, BTK mutation (*C481S*) is associated with secondary ibrutinib resistance and thus identifying this mutation longitudinally might be an effective strategy to save valuable time and to select other non-BTK options. Indeed, in the BTK resistant setting, several important anti-apoptic (such as *BCL-2*) and B-cell pathways (*PI3K-AKT*)/ (alternative NF-KB) might be up-regulated. The *BCL-2* inhibitor ABT-199 achieved impressive response in CLL and studies in MCL are actively pursuing treatment options with ABT-199 which might provide a treatment breakthrough. Translational models should be developed so that these alternative survival mechanisms can be monitored prospectively. These alternative pathways can be targeted in the context of drug resistance and such data may guide clinical decision making. We have developed first patient derived xenograft (PDX) mouse model in MCL previously [70] that used primary cells collected from patients injected into a fetal bone chip that provided the tumor microenvironment. The PDX model provided a suitable platform to test drug efficacy *in vivo* and also has excellent clinical potential to guide therapeutic choice [71]. We are currently initiating the first adaptive trial in MCL based on the PDX platform and an exploratory objective of the trial is to evaluate mutations before and after treatment. This would allow assessing the impact of gene mutations on success and failure of novel therapies in MCL and may help unblock the drug-resistance bottleneck.

It is apparent from our review, that majority of the somatic mutations described by the studies were either at diagnosis or pre-treatment phase with only a smaller subset of patients being relapsed/refractory. Thus, future works should assess mutations among treatment refractory patients to better understand the correlation of postulated survival pathways and clinical heterogeneity. Stratifying MCL patients by tumor mutations would require validation from further studies that include diverse sub-groups of patients. The clinical trials that targeted specific mutation previously (such as flavopiridol, HDAC inhibitors) have not led to major improvement in overall survival. With progression of the disease, one sees increasing number of genetic aberrations that was not present originally. Thus, mutations that were present at diagnosis or pre-treatment should be compared with mutations at disease progression. These analysis could identify important compensatory, pro-survival and anti-apoptic pathways and actionable genetic targets.

## CONCLUSIONS

Increased reporting of somatic mutations opened up a great opportunity to profile frequent genetic lesions in MCL. A combinatory approach with frontline agents and agents that target specific genetic aberration might be helpful for durable remission. Since multitudes of drugs are currently available that target different components of the cell signaling pathways, dynamic adaptive trials that could promptly assess patients' genetic aberrations and potentially switch treatment is the next cornerstone to combat drug resistance in MCL.
